# Network pharmacology analysis and experimental validation to explore the mechanism of kaempferol in the treatment of osteoporosis

**DOI:** 10.1038/s41598-024-57796-3

**Published:** 2024-03-26

**Authors:** Qi Dong, Guoxia Ren, Yanzhao Li, Dingjun Hao

**Affiliations:** 1 Department of Orthopedics, The First Affiliated Hospital of Xi’an Jiaotong University, Xi’an, Shaanxi China; 2 Department of Physical Medicine and Rehabilitation, The Affiliated Hospital of Shaanxi University of Chinese Medicine, Xianyang, Shaanxi China; 3 Department of Physical Medicine and Rehabilitation, Xi’an Chest Hospital, Xi’an, Shaanxi China; 4 Department of Traditional Chinese Medicine, First Clinical Medical College, Shaanxi University of Chinese Medicine, Xianyang, Shaanxi China

**Keywords:** Kaempferol, Osteoporosis, Network pharmacology, Molecular docking, In vitro validation, Drug discovery, Pharmacology, Pharmacogenetics

## Abstract

Osteoporosis (OP) is a prevalent global disease characterized by bone mass loss and microstructural destruction, resulting in increased bone fragility and fracture susceptibility. Our study aims to investigate the potential of kaempferol in preventing and treating OP through a combination of network pharmacology and molecular experiments. Kaempferol and OP-related targets were retrieved from the public database. A protein–protein interaction (PPI) network of common targets was constructed using the STRING database and visualized with Cytoscape 3.9.1 software. Enrichment analyses for GO and KEGG of potential therapeutic targets were conducted using the Hiplot platform. Molecular docking was performed using Molecular operating environment (MOE) software, and cell experiments were conducted to validate the mechanism of kaempferol in treating OP. Network pharmacology analysis identified 54 overlapping targets between kaempferol and OP, with 10 core targets identified. The primarily enriched pathways included atherosclerosis-related signaling pathways, the AGE/RAGE signaling pathway, and the TNF signaling pathway. Molecular docking results indicated stable binding of kaempferol and two target proteins, AKT1 and MMP9. In vitro cell experiments demonstrated significant upregulation of *AKT1* expression in MC3T3-E1 cells (*p* < 0.001) with kaempferol treatment, along with downregulation of *MMP9* expression (*p* < 0.05) compared to the control group. This study predicted the core targets and pathways of kaempferol in OP treatment using network pharmacology, and validated these findings through in vitro experiments, suggesting a promising avenue for future clinical treatment of OP.

## Introduction

Osteoporosis (OP) is a systemic bone disease characterized by reduced bone mass and degeneration of bone microstructure, leading to increased bone fragility and the risk of fractures^1^. OP is a global health problem, especially among the elderly. According to statistics, its prevalence worldwide signifies a pervasive threat to the health of older individuals. This not only results in severe pain and extended recovery periods but also hampers mobility, diminishes quality of life, and elevates the risk of mortality^2^.According to epidemiological data released by the National Health Commission of China in 2018, the prevalence of OP among individuals aged over 50 in China was 19.2%, comprising 6.0% of males, 32.1% of females, 16.2% in urban areas, and 20.7% in rural areas. As China’s population continues to age, the projected estimate of affected individuals is anticipated to surge to 212 million by 2050, emphasizing OP as a substantial public health concern^3^. OP arises from an imbalance between bone resorption and formation, relying on the coordination of osteoclasts and osteoblasts^4^. In OP patients, bone resorption outpaces formation, resulting in decreased bone mass and strength^5^. Various molecular signaling pathways contribute to OP development, including RANK/RANKL/OPG, Wnt/β- catenin, and estrogen signaling pathway, crucial in regulating osteoclasts and osteoblasts activity. RANKL (osteoprotegerin ligand) activates the formation and activation of osteoclasts by binding to its receptor, thus promoting bone absorption. OPG (osteoprotegerin) is a natural antagonist of RANKL, which can bind to RANKL and prevent it from binding with RANKL, thus inhibiting bone resorption^6^. Activating the Wnt/β- catenin pathway can promote the proliferation and differentiation of osteoblasts and increase bone formation^7,8^. Estrogen can inhibit bone resorption by reducing the formation of osteoclasts and prolonging their life span, and can also promote the activity of osteoblasts^9^. Notably, strategies for OP treatment focus on slowing bone resorption or promoting bone formation, employing options like anti-bone resorption medications (bisphosphonates, SERMs, and PTH analogs) and bone formation promoters (abaloparatide, denosumab, and romosozumab)^10–12^. However, these treatments face challenges due to potential side effects and the limitation of targeting a single molecular target^4,13–15^, necessitating comprehensive approaches to address OP’s intricate pathophysiology. Moreover, a combination of suitable exercise, adequate calcium, and vitamin D remains a crucial strategy for OP prevention and treatment^16^. Nonetheless, for some elderly patients, these interventions alone may be insufficient^17^, and the drugs currently used in clinical practice lack specificity. Therefore, there is an urgent need to explore novel approaches to address this pressing issue.

OP is categorized in Traditional Chinese Medicine (TCM) as “bone impotence”, “bone paralysis”, and “kidney deficiency”, with its key pathogenesis attributed kidney deficiency. Currently, the use of kidney-tonifying Chinese medicine in OP treatment has become a research focus. Moreover, the active components of Chinese herbal medicine, including Kaempferol, a flavonoid found in several Chinese herbs, hold significant research value and application prospects in OP treatment. In vitro and in vivo experiments conducted by numerous researchers have demonstrated the anti-OP effects of kaempferol and kaempferol-containing plants. These studies reveal that kaempferol plays a pivotal role in treating OP by inhibiting adipogenesis, inflammation, oxidative stress, osteoclast autophagy, and osteoblast apoptosis, while simultaneously activating osteoblast autophagy^18–23^. Consequently, kaempferol holds excellent clinical application prospects and can serve as a complementary or alternative treatment for OP.

Although there have been reports on the use of kaempferol in the treatment of OP, the underlying mechanism of action has not been investigated using network pharmacology. In view of this, this study utilized network pharmacology analysis and molecular docking technology to systematically analyze the potential mechanism of kaempferol against OP, with further validation through in vitro cell experiments. This objective of this study is to provide insights into the mechanism of kaempferol in the treatment of OP.

## Materials and methods

**Figure 1:**
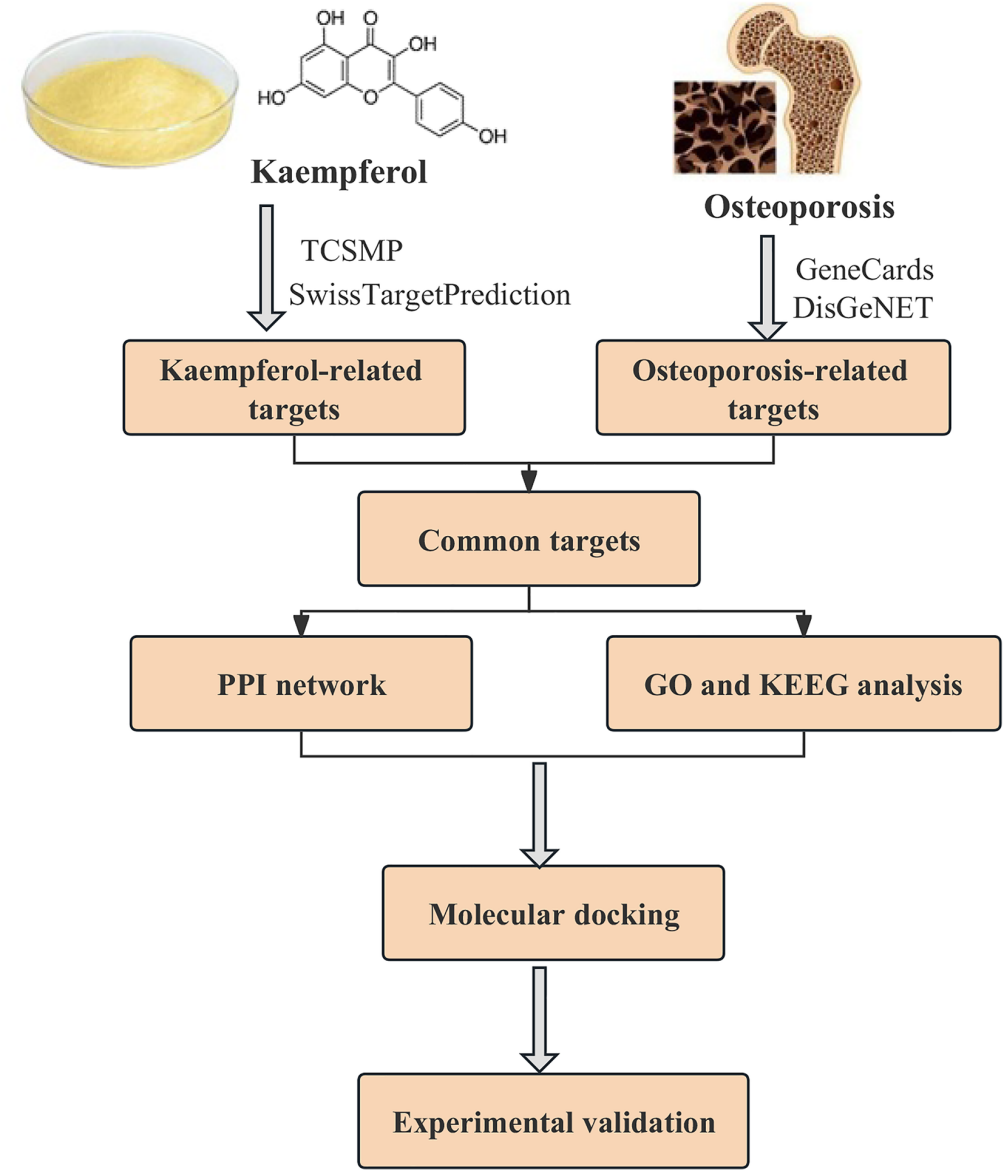
Whole framework of network pharmacology analysis.

The basic framework of the study is depicted in Fig. 1.

### Extraction of target genes

Kaempferol-related targets were predicted using the TCMSP (https://old.tcmsp-e.com/index.php)^24^ and SwissTargetPrediction (http://www.swisstargetprediction.ch/) databases^25^. Following screening, the targets were standardized by inputting them into the UniProt database (https: //www.uniprot.org)^26,27^ to obtain the official names of the targets.

### Screening of OP-related targets

Using “osteoporosis” as the keyword and “Homo sapiens” as the species, OP-related targets were collected from the GeneCards (https://www.genecards.org/)^28^ and DisGeNET (https://www.disgenet.org/) databases^29^. The intersection of the two databases was then considered to determine the OP-related targets.

### Acquisition of kaempferol-OP common targets and construction of protein–protein interaction (PPI) network

The intersection of kaempferol-related targets and OP-related targets was obtained using the R software (v.4.2.2) package “VennDiagram” (v.1.7.3) through Hiplot Pro (https://hiplot.com.cn/)^30^. The common targets obtained were then imported into the STRING database (https://stringdb.org/)^31^, and the PPI network was constructed with “Homo sapiens” as the species. Subsequently, the data were imported into Cytoscape 3.7.2 software for visual analysis, and cytoHubba plug-in was utilized to identify the core targets based on the top 10 rankings in maximal neighborhood component (MNC), node connect degree (Degree) and node connect closeness (Closeness).

### Gene ontology (GO) functional annotation and Kyoto Encyclopedia of Genes and Genomes (KEGG) enrichment analysis

The GO and KEGG enrichment analysis of the common targets was conducted using the R software (v.4.2.2) package clusterProfiler (v.4.5.0) through Hiplot Pro (https://hiplot.com.cn/)^30^, a comprehensive web service for biomedical data analysis and visualization. The results were presented in the form of bubble diagrams, bar diagrams, and clustering dendrograms, with significance denoted by *p* < 0.05.

### Molecular docking verification

Molecular docking, a crucial technique in network pharmacology analysis, involves combining known protein targets with small compounds to validate compound-target interactions. In this study, molecular docking analysis was performed using Molecular operating environment (MOE) software version 2019. The protein’s three-dimensional structure was retrieved from the Protein Data Bank (PDB) (http://www.rcsb.org/)^32^ and preprocessed by removing water molecules, preparing the protein structure, and minimizing the energy. The protein structure was then imported into MOE to construct a docking pocket. Small compound structures were obtained from PubChem (https://pubchem.ncbi.nlm.nih.gov/)^33^ and subjected to molecular docking analysis with the docking pocket.

### Experimental verification

#### Preparation of kaempferol solution

Kaempferol (purity = 99.86%) was purchased from MedChemExpress (MCE). The kaempferol dry powder was dissolved in dimethyl sulfoxide (DMSO) to a concentration of 10 mM and stored at -20℃ for subsequent use.

#### Cell culture

Pre-osteoblastic MC3T3-E1 cells were purchased from Procell Life Science&Technology Co., Ltd (Wuhan, China). The cells were cultured in α-modified Eagle’s medium (α-DMEM; Thermo, MA) supplemented with 10% fetal bovine serum (FBS), 100 U/mL penicillin, and 100 μg/mL streptomycin. Simultaneously, the cells were seeded into a cell culture plate and incubated at 37 °C in a humidified atmosphere with 5% CO_2_.

#### The effect of kaempferol on cell viability

Cell viability was assessed using cell counting kit-8 (CCK-8) assays following the manufacturer’s instructions. Initially, MC3T3-E1 cells were seeded in 6-well plates at a density of 5 × 10^3^ cells/well and cultured for 24 h at 37 °C with 5% CO_2_. Subsequently, various concentrations (2.5, 5, 7.5, 10, 12.5, and 15 μM) of kaempferol were added to the cells. After 24 and 48 h, the cells were treated with 10 μL CCK-8 solution for 4 h at 37℃, and the optical density (OD) at 450 nm was measured by a microplate reader.

#### Total RNA extraction and real-time quantitative polymerase chain reaction (RT-qPCR)

The MC3T3-E1 cells were inoculated into 6-well cell culture plates when they reached a healthy growth condition. Upon reaching 90% cell density, various concentrations of kaempferol (5 μM and 7.5 μM) were added, determined based on the cell viability assay. After 24 h, total RNA was extracted using TRIzol reagent (Sigma, USA), and the RNA concentration was measured with NanoDrop 2000 (Thermo, USA). Reverse transcription was performed using the TaKaRa reverse transcription kit (TaKaRa, Japan). GAPDH served as an internal reference, and the mRNA expression levels of *AKT1* and *MMP9* in each group were assessed using an ABI 7500 PCR instrument (Thermo, USA). Additionally, the relative mRNA expression levels were calculated using the 2^-△△Ct^ method. Each sample was assayed at least three times. The primer sequences for PCR were as follows:

AKT1: 5′-GGACTACTTGCACTCCGAGAAG-3′, 3′-CATAGTGGCACCGTCCTTGATC-5′;

MMP9: 5′-GCTGACTACGATAAGGACGGCA-3′, 3′-TAGTGGTGCAGGCAGAGTAGGA-5′;

GAPDH: 5′-GAGTCAACGGATTTGGTCGT-3′, 3′-GACAAGCTTCCCGTTCTCAG-5′.

#### Western blotting

The cells were inoculated and treated as described above. After 24 h of kaempferol treatment, total proteins were extracted and quantified using the BCA quantitative kit (Beyotime, China). Subsequently, they underwent polyacrylamide gel electrophoresis (PAGE). The proteins were then transferred to a polyvinylidene fluoride (PVDF) membrane, which was sealed with 5% skim milk. The membrane was incubated with primary antibodies and horseradish peroxidase (HRP) -conjugated secondary antibodies. Enhanced Chemiluminescence (ECL) was employed for protein visualization, and images were captured using the XLI Touch Imager (e-BLOT, China). Each band was measured thrice, and results are expressed as the mean ± standard deviation (SD). Glycerol 3- phosphate dehydrogenase (GAPDH) served as the internal control. Primary antibodies used in protein blot analysis were anti-AKT1 (1: 750, ZEN BIO, China), anti-MMP9 (1: 1500, Proteintech, China) and anti-GAPDH (1: 20,000, Proteintech, China).

#### Statistical analysis

The statistical analysis of experimental data was performed using GraphPad Prism v.9.0.0 software. All measurement data are presented as mean ± SD. Group comparisons were conducted using one-way ANOVA and two-way ANOVA for multiple groups. The experimental data were derived from three independent experiments.

## Results

### Information of kaempferol

**Figure 2:**
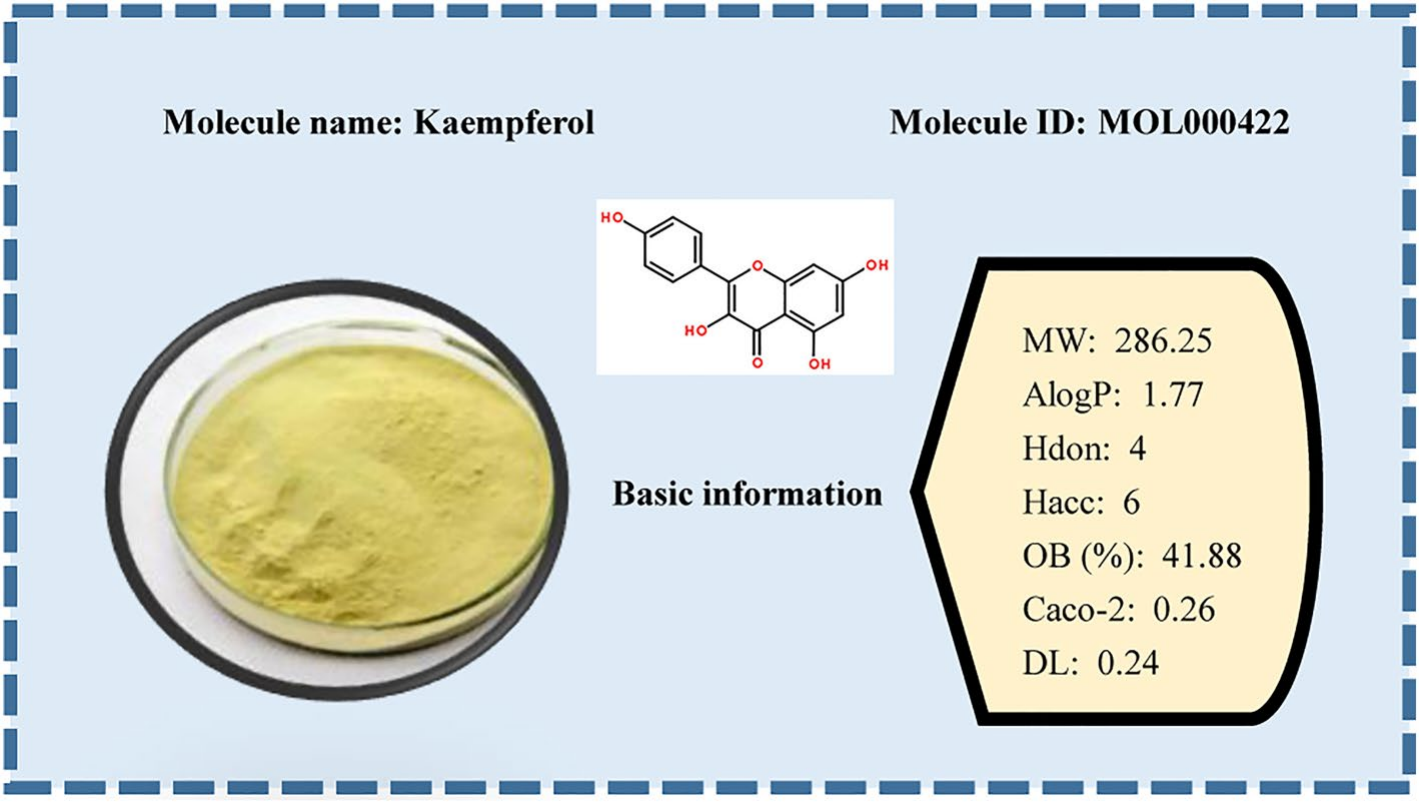
Diagram of the basic information of kaempferol.

The structure and other basic information about kaempferol are illustrated in Fig. 2. Notably, its oral bioavailability (OB) exceeded the threshold of 30% at 41.88%, while its drug-likeness (DL) value of 0.24 also surpassed the threshold of 0.18. These findings suggest that kaempferol exhibits promising potential for drug activity.

### Kaempferol-related targets

**Figure 3:**
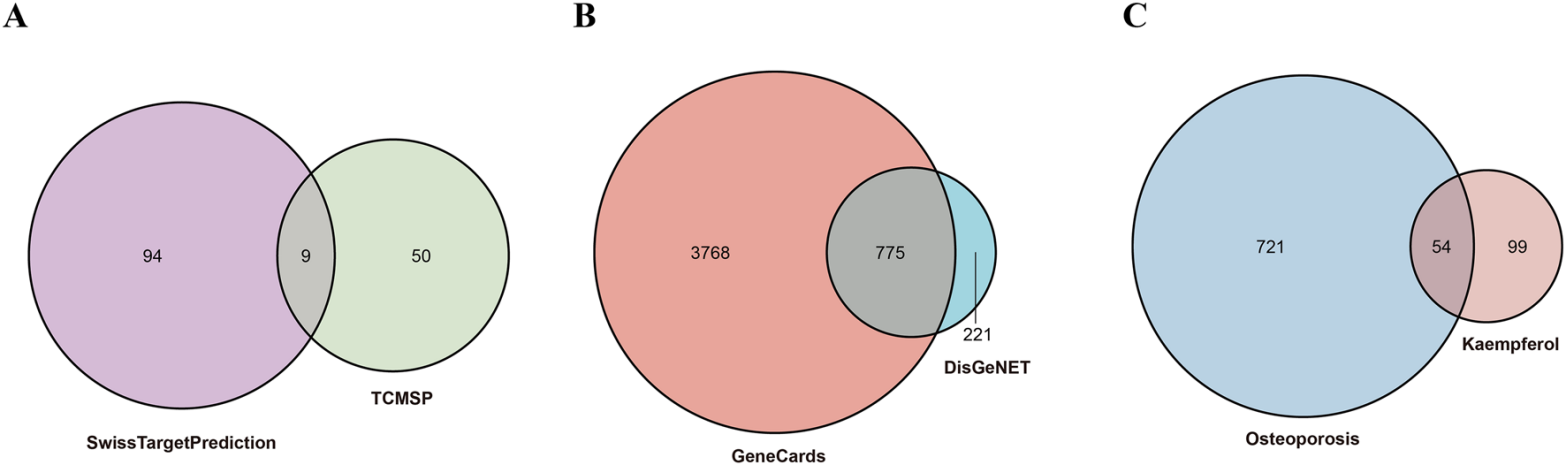
Venn diagram of kaempferol related targets (**A**), OP related targets (**B**), and targets shared by both (**C**).

A total of 59 kaempferol-related targets were obtained from the TCMSP database, while 103 related targets were retrieved from the SwissTargetPrediction database. After removing duplicates, a final dataset of 153 kaempferol-related targets was compiled by merging the data from both the TCMSP and SwissTargetPrediction databases (Fig. 3A).

### OP-related targets and overlapping targets

A total of 4543 OP-related targets were retrieved from the GeneCards database, with an additional 996 targets were obtained from the DisGeNET database. By intersecting these two datasets, a final set of 775 OP-related targets was generated (Fig. 3B). In total, 54 overlapping targets of kaempferol and OP wereidentified through the Venn diagram on the Hiplot platform, as depicted in Fig. 3C. These overlapping targets of kaempferol and OP may signify potential targets of kaempferol in the treatment of OP.

### PPI network construction

**Figure 4:**
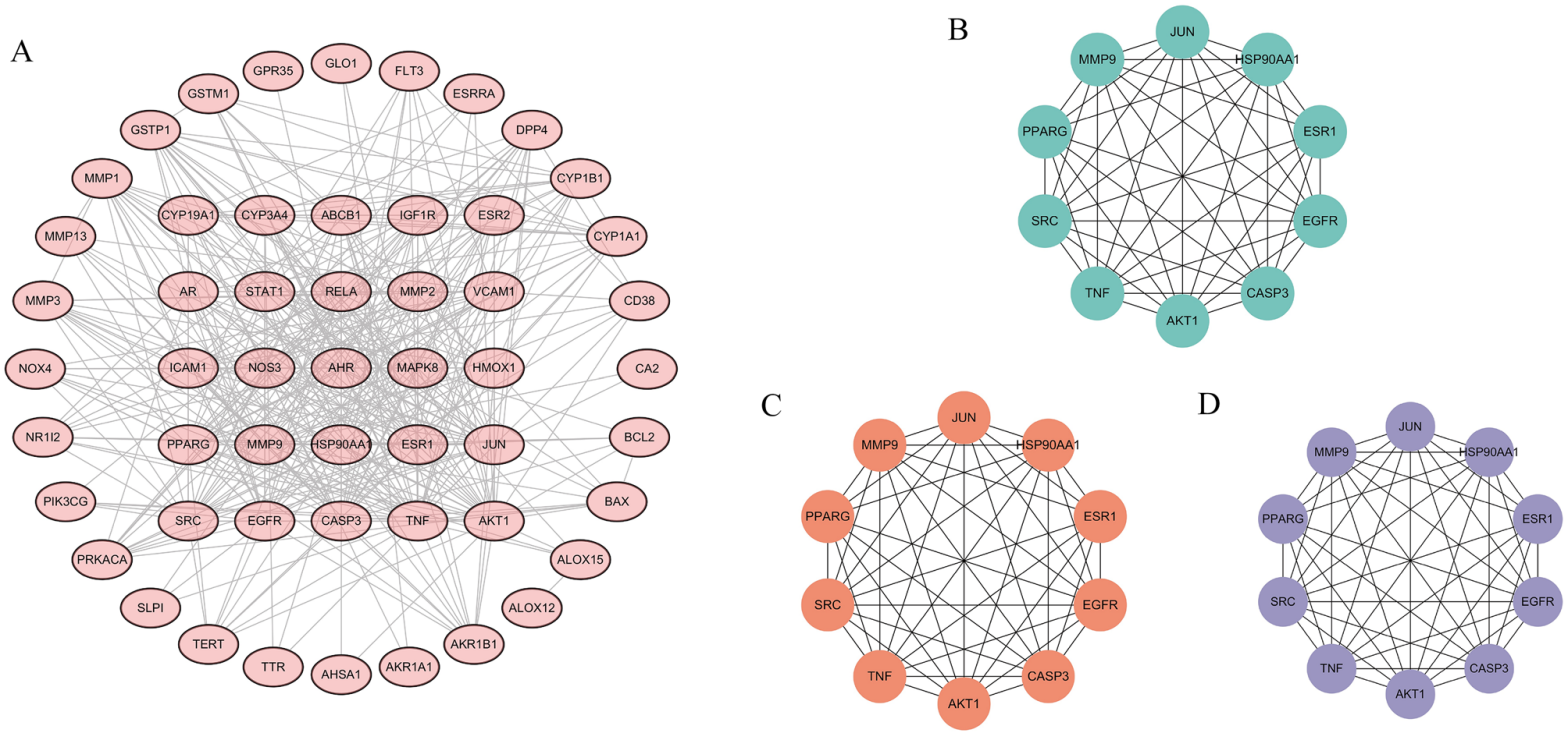
(**A**) PPI network construction. Core target network based on MNC (**B**), Degree (**C**) and Closeness (**D**) parameters.

The above overlapping targets of kaempferol and OP were input into the STRING database to construct the PPI network. Subsequently, the overlapping targets were imported into Cytoscape 3.10.0 for visualization, and the resulting PPI network is displayed in Fig. 4A. The cytoHubba plug-in was utilized to identify the core targets based on the top 10 rankings in MNC, Degree, and Closeness. Notably, the top 10 targets based on the MNC (Fig. 4B), Degree (Fig. 4C), and Closeness (Fig. 4D) parameters consistently included *AKT1*, *MMP9*, *ESR1*, *TNF*, *HSP90AA1*, *PPARG*, *SRC*, *EGFR*, *JUN*, and *CASP3*.

### GO functional annotation and KEGG signaling pathway enrichment analysis

**Figure 5:**
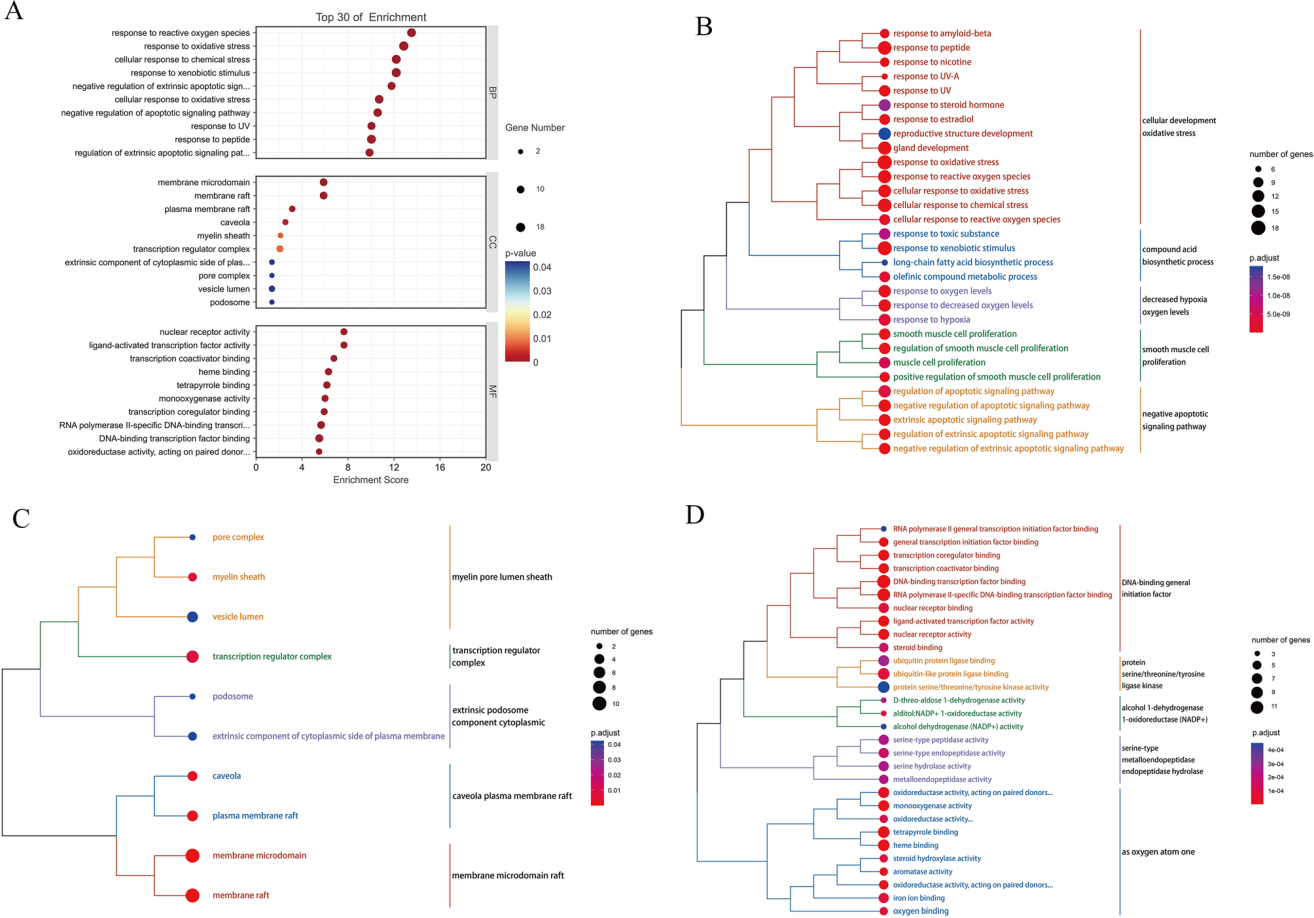
(**A**) Bubble plot of GO enrichment analysis. Cluster dendrogram of GO enrichment analysis based on BP (**B**), CC (**C**), and MF (**D**) classifications.

The Hiplot platform was utilized to conduct GO functional annotation analysis on 54 kaempferol-OP overlapping targets (*p* < 0.05). The GO enrichment analysis included three categories: biological process (BP), cellular components (CC), and molecular function (MF). Figure 5A illustrates the top 10 enrichment items for each category, ranked according to their *p-*values. In the BP section, the overlapping targets demonstrated enrichment in cellular development oxidative stress, including response to reactive oxygen species, response to oxidative stress, and cellular response to chemical stress (Fig. 5A,B). In the CC section, enrichment was observed in membrane microdomain raft structures, such as membrane raft and membrane microdomain (Fig. 5A,C). Furthermore, the common targets in the MF section were associated with DNA-binding general initiation factors, such as nuclear receptor activity and ligand-activated transcription factor activity (Fig. 5A,D).

**Figure 6:**
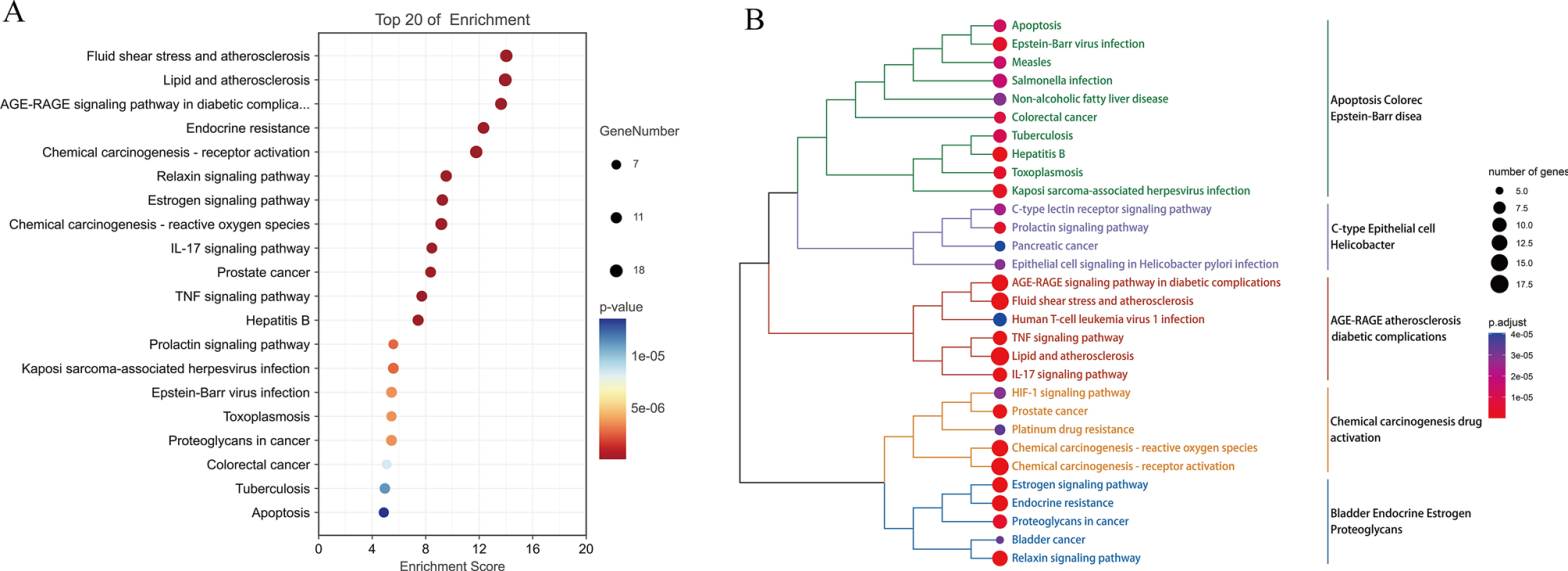
Bubble plot (**A**) and cluster dendrogram (**B**) of KEGG enrichment analysis.

KEGG signaling pathway enrichment analysis was performed on 54 kaempferol-OP overlapping targets (*p* < 0.05) using the Hiplot platform. Figure 6A displays the top 20 signaling pathways identified. The analysis revealed that the overlapping targets were enriched in several pathways related to AGE-RAGE atherosclerosis diabetic complications. These pathways include Fluid shear stress and atherosclerosis, Lipid and atherosclerosis, AGE-RAGE signaling pathway in diabetic complications, IL-17 signaling pathway, and TNF signaling pathway (Fig. 6A,B).

### Molecular docking analysis

Among the ten core targets of kaempferol for OP treatment, *AKT1*, and *MMP9* were chosen for molecular docking. The decision to select *AKT1* and *MMP9* as molecular docking targets is based on two primary reasons. Firstly, *AKT1* demonstrates the highest Degree value within the PPI network, signifying its importance in the network. Secondly, *AKT1* and *MMP9* are both enriched in multiple signaling pathways, such as Fluid shear stress and atherosclerosis, Lipid and atherosclerosis, AGE-RAGE signaling pathway in diabetic complications, and TNF signaling pathway.

**Figure 7:**
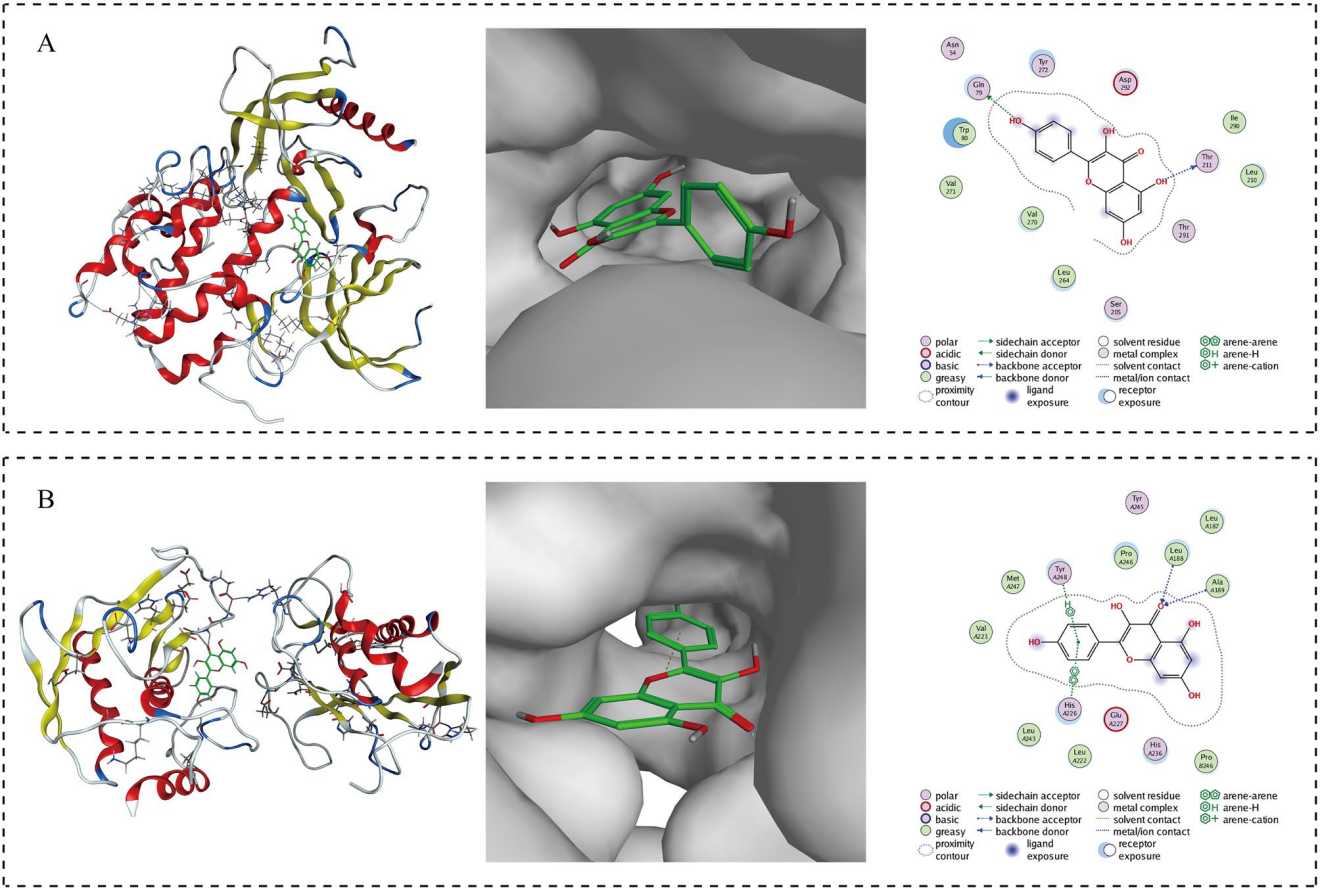
Simulated molecular docking of kaempferol on *AKT1* (**A**) and *MMP9* (**B**).

To explore the binding of kaempferol with these two targets, MOE was utilized to predict their interactions. The binding energy, indicating the strength of the interaction, was assessed in this study. Generally, a binding energy of less than 0 kcal mol^−1^ suggests binding activity between molecules, while a binding energy of less than − 5.0 kcal mol^−1^ indicates strong binding activity. The smaller the binding energy, the stronger the binding ability. The results of molecular docking analysis of kaempferol with *AKT1* and *MMP9* can be observed in Fig. 7. The binding energies were calculated to be 5.89 kcal mol^−1^ and 6.51 kcal mol^−1^, respectively. These values are below − 5 kcal mol^−1^, indicating that kaempferol exhibited significant and strong binding ability with both *AKT1* and *MMP9*.

### Effects of kaempferol on the viability of MC3T3-E1 cells

**Figure 8:**
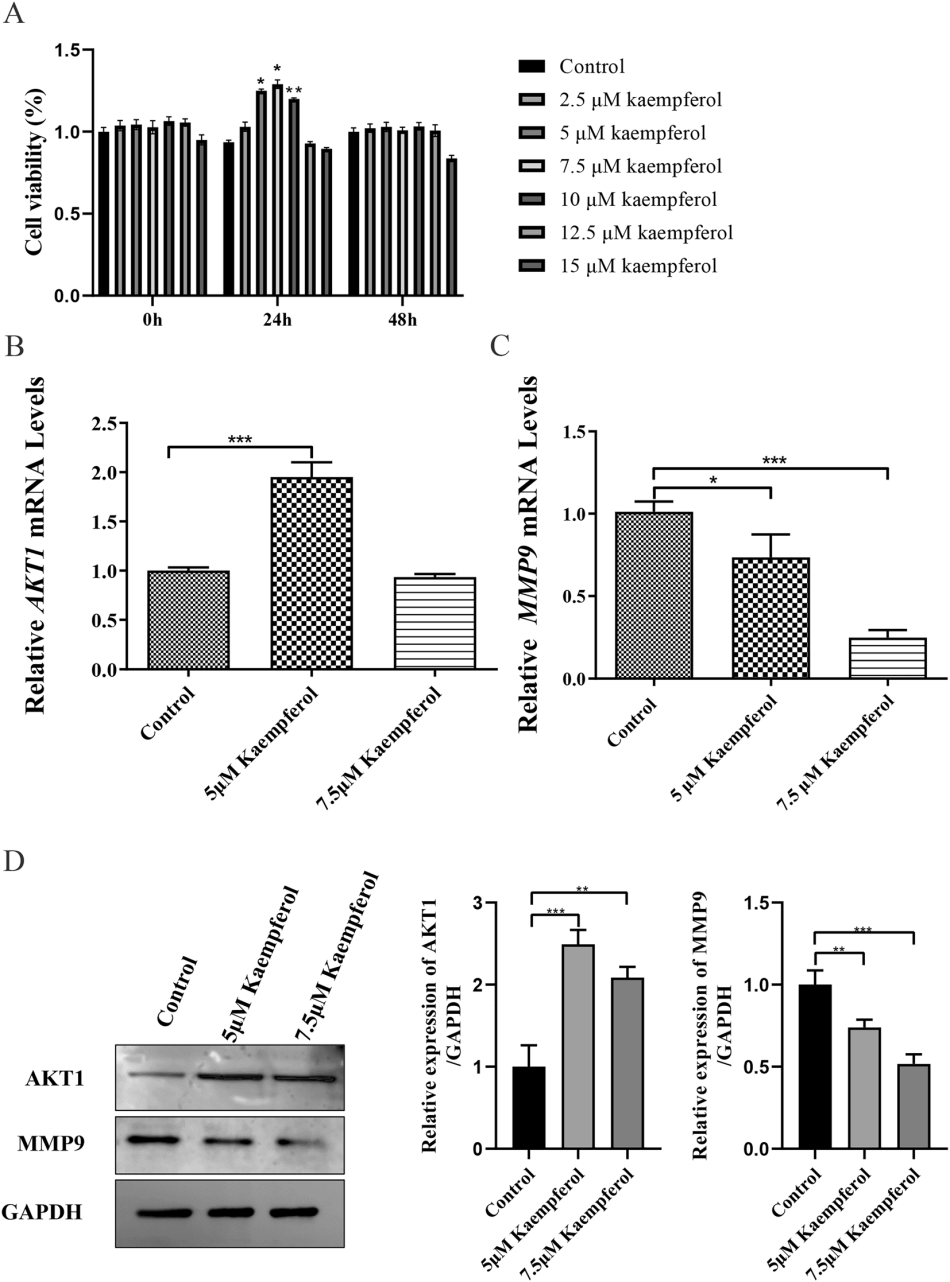
Kaempferol affects the expression of *AKT1* and *MMP9* expression. (**A**) Results of cell viability assay. The statistical analyses were conducted by two-way ANOVA. * *p* < 0.05, ** *p* < 0.01 vs. control group. (**B**, **C**) Quantitative analysis of *AKT1* and *MMP9* mRNAs. (**D**) The analysis of the protein expression of AKT1 and MMP9. * *p* < 0.05, ** *p* < 0.01, *** *p* < 0.001. The statistical analyses were conducted by one-way ANOVA. Results are shown as mean ± SD of three individual experiments.

After treating MC3T3-E1 cells with various concentrations of kaempferol (2.5, 5, 7.5, 10, 12.5, and 15 μM) for 24 h and 48 h, we observed that the concentrations of 5 μM and 7.5 μM of kaempferol had the most significant impact on the activity of MC3T3-E1 cells at 24 h (*p* ˂ 0.05) compared to the control group (Fig. 8A). When MC3T3-E1 cells were co-cultured with kaempferol concentrations of 5 μM and 7.5 μM for 24 h, the drug significantly promoted cell viability (*p* < 0.05 and *p* < 0.01, respectively). Therefore, MC3T3-E1 cells treated with 5 μM and 7.5 μM of kaempferol for 24 h were selected for further experiments.

### Effects of kaempferol on the expression of *AKT1* and *MMP9* in MC3T3-E1 cells

In Fig. 8B, the mRNA expression of *AKT1* in MC3T3-E1 cells exhibited a significant increase (*p* < 0.001) when treated with 5 μM of kaempferol compared to the control group. However, under 7.5 μM of kaempferol, no significant effect was observed on the expression of *AKT1* (*p* > 0.05). Conversely, the mRNA expression of *MMP9* in MC3T3-E1 cells significantly decreased (*p* < 0.05) under both 5 μM and 7.5 μM kaempferol (Fig. 8C). We further analyzed the protein expression of AKT1 and MMP9. As depicted in Fig. 8D, the protein expression of AKT1 was increased in the dosing group compared with that in the control group, while the protein expression of MMP9 was decreased in the dosing group compared to the control group. These findings suggest that kaempferol may play an anti-OP role by up-regulating the expression of *AKT1* and down-regulating the expression of *MMP9*.

## Discussion

Network pharmacology is a novel research methodology that combines systems network analysis with pharmacology to elucidate the potential mechanisms of multi-target drugs in the molecular-level treatment of diseases through compound-target and target-disease network analyses^34–36^. The present study employed a network pharmacology approach to investigate the mechanism of action underlying the therapeutic use of kaempferol in treating OP. The findings identified 54 potential therapeutic targets for kaempferol in OP treatment, with *AKT1*, *MMP9*, *ESR1*, *TNF*, *HSP90AA1*, *PPARG*, *SRC*, *EGFR*, *JUN*, and *CASP3* identified as the core targets. Moreover, KEGG enrichment analysis indicated that the overlapping targets of kaempferol and OP were predominantly associated with signaling pathways implicated in atherosclerosis, the AGE/RAGE signaling pathway, and the TNF signaling pathway. By integrating the PPI network (with *AKT1* having the highest degree value), KEGG signaling pathway enrichment analysis (which identified *AKT1* and *MMP9* as being enriched in multiple signaling pathways, including those associated with atherosclerosis, AGE-RAGE, and TNF, all of which are closely linked to OP), and molecular docking analysis (which revealed the significant and strong binding ability of kaempferol to *AKT1* and *MMP9*), it becomes apparent that *AKT1* and *MMP9* play a crucial role in the therapeutic effects of kaempferol against OP.

Exploring the key targets of kaempferol in treating OP is crucial for elucidating its potential mechanism of action. This study highlights the importance of regulating the expression of *AKT1* and *MMP9* in OP treatment. RAC-alpha serine/threonine-protein kinase (AKT1) is a protein kinase present in osteoblasts and osteoclasts that plays a crucial role in regulating cell proliferation, differentiation, and maintaining bone mass. Studies have shown that a deficiency of *AKT1* can lead to bone loss, which is associated with OP^37^. Yanjun Wang et al. demonstrated that tanshinone may improve OP by targeting and up-regulating *AKT1* expression^38^. Li Ou et al. found that *Rehmanniae Radix Preparata* could increase bone density and promote bone formation by significantly increasing *AKT1* expression in bone tissue^39^, highlighting the significance of *AKT1* in bone health. Matrix metalloproteinase 9 is a Zn^2+^-dependent proteolytic enzyme that is closely related to the pathogenesis of OP. Evidence shows that serum MMP-9 concentration is negatively correlated with bone mineral density, which is considered a biochemical marker of bone resorption and reconstruction, and an important marker for early diagnosis of OP^40,41^. Additionally, Guoju Hong et al. showed that Robinin may inhibit osteoclast proliferation to prevent OP by down-regulating the expression of the osteoclast target gene, *MMP9*^42^. Consistent with our cell experiments, kaempferol significantly increased the expression of *AKT1* and decreased the expression of *MMP9*. Therefore, we hypothesize that the therapeutic effect of kaempferol in OP may be attributed to its ability to modulate the expression levels of *AKT1* and *MMP9*.

KEGG enrichment analysis can identify pathways and functions related to specific diseases or physiological processes, offering insights into potential candidate targets. Our study revealed that common targets of kaempferol and OP are mainly involved in the Atherosclerosis-related signaling pathways, AGE/RAGE signaling pathway, and TNF signaling pathway. Epidemiological studies have demonstrated a strong association between atherosclerosis and OP, suggesting they may share a common pathogenic mechanism^43,44^. This explains why the common targets of kaempferol and OP are enriched in multiple atherosclerosis-related signaling pathways. Concurrently, numerous studies have shown that the AGE‐RAGE interaction can induce osteoblast apoptosis, inhibit cell proliferation and migration, decrease bone mass, and promote OP in diabetic patients^45^. Evan G Buettmann et al.^46^ concluded that bone loss due to aging and disuse is associated with AGE-RAGE signaling pathway. Liang Mo et al.^47^ found that the AGE-RAGE signaling pathway in diabetic complications may play a significant role in diabetic skeletal fragility, including bone function impairment. Additionally, other clinical symptoms of diabetes, such as decreased bone mineral density, inhibition of bone turnover markers, and bone quality impairment, are all closely related to the AGE-RAGE signaling pathway^48^. Among the KEGG enrichment results, the TNF signaling pathway was also significantly enriched. TNF has been reported to have a significant impact on bone metabolism, aligning with previous studies^49,50^. Moreover, TNF-α, primarily produced by activated mononuclear macrophages, is closely associated with OP, with high expression levels detected in OP cases^51^. Importantly, the key targets *AKT1* and *MMP9* are also enriched in the aforementioned signaling pathways. Therefore, we propose that kaempferol’s treatment for OP exhibits a multi-pathway effect and might improve OP by regulating the expression of relevant genes, thereby influencing the associated pathways.

The limitation of this study is that the predicted results were mainly validated through cell experiments, and further studies involving animal and clinical trials are lacking. Further exploration is needed to better understand the mechanism of kaempferol in treating OP, thereby strengthening the persuasiveness of our findings.

## Conclusion

In conclusion, our study preliminarily elucidated that kaempferol exhibited characteristics of targeting multiple pathways in the treatment of OP, as evidenced by network pharmacology analysis and in vitro experiments. The mechanism of action of kaempferol in treating OP may involve up-regulating the expression of *AKT1* and down-regulating the expression of *MMP9*, while participating in the Atherosclerosis-related signaling pathways, AGE/RAGE signaling pathway, and TNF signaling pathway. This study contributes to the understanding of the mechanism of kaempferol in treating OP and provides a theoretical foundation for future research and development of new drugs.

## Data Availability

The datasets generated and analyzed during the current study can be found in the Kaempferol-related targets were predicted by the TCMSP (https://old.tcmsp-e.com/index.php) and SwissTargetPrediction (http://www.swisstargetprediction.ch/) database. After screening, targets were inputted into the UniProt database (https: //www.uniprot.org) for standardization to obtain the official names of the targets. With “OP” as the key word and “Homo sapiens” as the species, OP-related targets were collected from the GeneCards (https://www.genecards.org/) and DisGeNET (https://www.disgenet.org/) databases.
